# Agential realism as an alternative philosophy of science perspective for quantitative psychology

**DOI:** 10.3389/fpsyg.2024.1410047

**Published:** 2024-11-13

**Authors:** Julia Scholz

**Affiliations:** Institute of Psychology, University of Wuppertal, Wuppertal, Germany

**Keywords:** agential realism, philosophy of science, intra-action, phenomena, agential cut, replication, ethico-onto-epistemology, realization potential

## Abstract

This paper introduces Karen Barad’s philosophical framework of agential realism as an alternative philosophy of science perspective for quantitative psychology and measurement. Agential realism offers a rethinking of the research object, measurement process and outcome, causality, and the researcher’s responsibility by proposing an ethico-epistem-ontological understanding of material-discursive practices that co-construct our world. The contemporary, canonical underlying philosophy of science perspective of quantitative psychology entails entity realism, a difference between ontic existence and epistemic approaches, complete causality, and determinism. Consequently, the researcher has no responsibility for the characteristics of a research object. The paper introduces agential realism and its assumptions about rejecting entity realism but a particular understanding of phenomena, the entanglement of ontic existence and epistemic approaches, and the researcher’s role in co-creating an outcome. A reworking of the concept of causality implies newly emerging possibilities for realizations. Subsequently, the paper addresses four consequences of applying agential realism in quantitative psychology. (1) If there is indeterminacy in every phenomenon, researchers do not search for one true score but assume a realization potential, which has implications for comparisons and replications. (2) If configurations are part of things-in-phenomena, then context does not work as a third variable; instead, all ‘parts’ are co-creators. This entanglement must be considered in replications instead of trying to eliminate its impact. (3) Agential realism encompasses the researchers’ responsibility to justify decisions made in a research project and to clarify ethics. (4) Overall, agential realism alters the research endeavor by asking new questions and interpreting research outcomes differently. Further directions point towards concrete tasks like methodological questions and the necessity within psychology to elaborate further on the conceptualizations initiated by Barad.

## 1 Introduction

Psychological science faces, once again, discussions about its knowledge acquisition. The discussions should be seen as a sign of quality: science is open to questioning. Some ‘crises’ in the field forced psychologists, alongside colleagues from other disciplines, to reconsider what their knowledge represents. Psychologists’ knowledge is usually aimed at describing, understanding, explaining, and sometimes changing human thought, feeling, experience, and behavior. Besides these knowledge fields, psychology is also concerned about how psychological knowledge is gained. Besides previous debates about experimental research logic (*cf.* Gergen, 2001), recently, epistemological, conceptual, and methodological challenges in psychological science practices are again discussed (*cf.* Hanfstingl et al., 2023). For instance, the Open Science Collaboration (2015) identified a low replication rate, but Gilbert et al. (2016) accused the project of underestimating it. In the face of such discussions—Nosek et al. (2022) named the 2010s ‘a decade of active confrontation’—many very sophisticated articles analyzed statistical and methodological problems, and researchers devised various solutions to increase the replicability of experimental results. However, methodological procedure is not the focus of this paper. I will not discuss improvements in accomplishing and processing the contemporary standard quantitative method. Instead, I will discuss a shift in the philosophy of science perspective for quantitative psychology, and consider its consequences, also for replication. First, I will look at the underlying basis of contemporary quantitative psychology, and then I will propose an alternative: Karen Barad’s agential realism.

Agential realism was extensively adapted in a wide range of fields. For example, Barad’s article ‘Posthumanist Performativity’ (2003) has already been cited more than 10.000 times. The book ‘Meeting the Universe Halfway’ (2007) more than 20.000 times. Hollin et al. (2017) give a little peek at Barad’s reception regarding content. However, there is only sparse reception within psychology, and if so, then primarily within qualitative approaches (e.g., Brown, 2020; Gemignani et al., 2023). Mauthner (2024) discusses broad changes within a research logic if we^1^ take an agential realist perspective but also brings qualitative methods to the fore. Besides my own work (Scholz, 2013, 2018), it was, for instance, Shotter (2014a) who encouraged psychologists to take Barad’s perspectives as a matter of principle. Next to a few discussions in the journal ‘Theory & Psychology’ (Højgaard and Søndergaard, 2011; Shotter, 2014b; Tobias-Renstrøm and Køppe, 2020), for example, Letiche et al. (2023) discussed agential realism for experiments and called for reworking of quality criteria of research. However, this was centered on ‘accounting’ and not directly about psychological experiments. Agential realism is hardly ever applied to quantitative research in psychology or questions of replicability. I will get back to this below, but first, I will look at the underlying philosophy of science perspective of quantitative psychology.

## 2 The underlying philosophy of science perspective of quantitative psychology

Every working paradigm has its foundational logic about why somebody is doing something, such as researchers having a reason to do science this way or that way. The starting points of every paradigm are pre-assumptions about the world from which methods are deduced. On the other hand, somebody who uses methods has pre-assumptions about the world in which the specific method makes sense. Psychology researchers typically do not explicate their foundational pre-assumptions in a research article, but these can mostly be read from the researcher’s proceeding or wording. Other pre-assumptions lead to a different proceeding or other wording. Regarding research logic, Popper’s ‘logic of scientific discovery’ is still widely used, though further developed and enhanced. I will mention where this logic plays a role in my argument but will not summarize it entirely. Instead, I will concentrate on the points still used in quantitative psychology but contrasted with the proposed alternative of agential realism. Table 1 offers shortened descriptions of conceptualizations from each perspective in a comparative manner.

**Table 1:** Comparison of concepts between the contemporary, canonical psychological, and the agential realist perspective.

Contemporary, canonical psychological perspective	Concept	Agential realist perspective
(No application)	Agential	The adjective is used to indicate that a correspondent referent (‘cut’, ‘realism’, or ‘separability’) does not exist *per se* but that an agency brings this referent into being
Bounded entity that is built of components and that can measure and/or manipulate something	Apparatus	Enacts intra-actions and agential cuts within the phenomenon; is itself entangled in material-discursive configurations
A vector of influence is transported from one process, state, or object to another process, state, or object	Causal	Intra-actions can enact a causal structure; causality is entangled with conditionality
bounded, determined occurrence with pre-existing properties/features (independent from a measurement process); can also be composed of several smaller components	Entity / object / relatum (here also: that subject matter of psychology which is referred to by nouns, like ‘neuron’, ‘sensibility’, ‘fear’ or ‘behavior’)	always unbounded as entity-within-phenomena or thing-within-phenomena or relatum-within-relations; does not exist without material-discursive configurations that, through intra-actions, enact this occurrence
Inter means between; interaction as an action between separate entities; separate entities influence each other or one entity influences the other	Interaction vs. intra-action	Intra means within or inside; intra-action as an action within an entanglement; intra-acting relata are not separate entities but relata-within-relations
Refers to having information about objective facts; if we ‘know’ something is an epistemological question	Knowing, knowledge	Knowing and being are mutually implicated: if we ‘know’ something is an onto-epistemological question
Material and discursive, or social, influences can impact a process, state, or object causally	Material-discursive configurations	Entangled configurations that enact intra-actions and set agential cuts; they situatedly co-create what exists
A (numerical) representation of a ‘real-world occurrence’	Measurement outcome	An occurrence enacted from intra-actions of material-discursive configurations
The structurally identical assignment of a numerical relative to an empirical one; an epistemic activity	Measurement process	An intra-action that enacts agential cuts; carves out one of several possibilities; co-creates characteristics of the research object
Independent of an onlooker, subject, or researcher	Objective	Accountive of the constitutive material-discursive configurations
A complex unit that entails different components and an inner structure	Phenomenon	An entangled non-bounded occurrence that can enact further agential cuts
Real is existent independent from an onlooker; reality refers to an objective world that exists independent of perceptions, beliefs, or thoughts	Real, reality	Something is real if there is a locally and temporarily shared ‘experience’ of intra-actions and cuts; not necessarily a human ‘experience’, can, for example, also be ‘experienced’ by physical radiation; reality is always a situated reality, dependent on material-discursive configurations;
Discovers what was already there	Research, science	Understands situated possibilities within material-discursive practices

In this text, I will, as Uher (2022) also urges, be sensitive to the distinction between ‘psychology’ and ‘psyche’, although many psychological texts do not make a clear distinction and use ‘psychology’ when referring to ‘psyche.’ However, since I will also address the approach of the discipline of psychology, I need to be clear in sentences whether I am talking about the discipline or the human psyche. Likewise, I will differentiate between ontic and ontological, as well as between epistemic and epistemological—find an overview of such differentiations in Table 2.

**Table 2:** Vocabulary differentiation in this text.

Epistemic	Refers to anything related to knowledge
Epistemological	Refers to anything related to the study or theory of knowledge
Ontic	Refers to anything related to being
Ontological	Refers to anything related to the study or theory of being
Psychic	Refers to anything related to the human psyche
Psychological	Refers to anything related to the study or theory of the human psyche
Onto-epistemical	Refers to anything related to the entanglement of being and knowing in the world
Onto-epistemological	Refers to anything related to the study or theory of the entanglement of being and knowing in the world

### 2.1 Entity realism

To start, I will examine the understanding of the constitution of the research objects within quantitative psychology. By ‘objects’ (Table 1), I mean the subject matter of psychology. It is that what is described with nouns in that discipline. These nouns can refer to physical things like ‘neuron’ or ‘lens’ but also to concepts like ‘self-confidence’ or ‘sensibility’, concrete experiences like ‘fear’, and broader categories like ‘behavior’ or ‘feeling.’ To compare philosophy of science perspectives, I will also use ‘entity’ (Table 1) for such a subject matter. Neurons, sensibility, fear, or behavior are all ‘entities’ and ‘research objects’ of the discipline of psychology.

Contemporary quantitative psychology comprises realism toward these research objects in that they are preexisting objects (i.e., individually determinate bounded entities) with inherent properties. This entity realism is one central assumption of the classic realist philosophy of science perspective. Dienes (2008) states some differing positions within psychology but closes that scientists need real entities to maintain a ‘subject matter.’ This aligns with Popper’s (2002) perspective, which puts a realist position not as a requirement for the ‘logic of scientific discovery’ but as the background in which the pursuit of truth gains meaning. Also, Herzog (2012) states that scientists classify themselves as belonging to what they call materialism or physicalism in a classical realist way. Some psychologists explicitly state that the research objects they investigate are not merely auxiliary constructs in an instrumentalist way but are ontologically (Table 2) understood as ‘real’ (Table 1). “Psychologists (…) also generally believe in the reality of the domain of their subjects—of mind, and brains, thoughts, images, networks, social pressures, social identities, psychological contexts and so on” (Dienes, 2008, p. 28). It is clear that psychological objects need not be physical objects (e.g., like a neuron) but can be a process, a state, a feeling, or the like. Uher (2021) also resumes that it is widespread for psychologists to ascribe an ontic status to constructs, which is entity realism. In a hypothesis like ‘increasing empathy reduces racial bias’, the constructs ‘empathy’ and ‘racial bias’ are assumed to exist before the researcher enters the stage. Therefore, I also use the terms ‘entity’ and ‘objects’ in this psychological realm for occurrences like ‘behavior’ or ‘emotion.’ The critical point is the philosophical pre-assumption about the occurrences that a discipline investigates, which can be physical objects in physics but might be behavior in psychology. In the following explanations, psychological entities and objects are not particles but ‘thoughts, images, networks’ and so on.

Researchers might acknowledge that such objects have developed over time (e.g., in the history of humanity), and they might call them ‘phenomena’ (instead of objects) to express that several components belong to such an item. However, in the moment of theorizing about and experimenting with such concepts like ‘empathy’, they assume each one is a particular set of parts (e.g., two persons) with their qualities (e.g., a feeling, a thought, a motive, an ability) and their relations to one another (e.g., one person has the ability to understand and possibly share a perspective or feeling of the other person). All such entities are assumed to be ‘real’ in that they exist individually and independently of any onlooker (Table 1).

### 2.2 A difference between ontic existence and epistemic approaches and the understanding of measurement

One basis of Popper’s (e.g., 2002) idea of scientific progress, which is still the foundation for quantitative psychology, is the *differentiation* between ontic existence and epistemic approaches to the existences. In this perspective, ‘ontic existence’ refers to what exists as concrete and factual nature of something and ‘epistemic approach’ refers to the tools we use to try to gain knowledge (Table 1) of the nature of our studied research objects. This differentiation is needed to assume that the ontic state of an object at any given time and place has a factual nature independent of researchers’ attempts to gain knowledge of such a factual nature. As Popper pointed out, the aim of science is (through falsification) to gain better and better descriptions and explanations of the (classic realist) objects and of the operating causal (Table 1) chains. Accordingly, our knowledge should grow in representations—as accurate as possible—of the real object or property of anything. In this perspective, the factual property or state of anything exists independent from our epistemic approaches but those approaches can be more or less suitable to deliver good representations of the factual property. Accordingly, researchers can have varying degrees of optimism about how close they might come to an ‘as accurate as possible’ or a ‘true’ representation of their research objects. However, suppose they conclude that the representations are too poor. In that case, this is attributed to epistemic reasons: the method was unsuitable, or flaws within the research design disturbed the measuring. The basic assumption of quantitative research is that, in principle, the research activity does not change the research object. If a research outcome does indicate a change in the factual nature, this must be an error. Such an indication can only demonstrate that the epistemic approach was unsuitable because ontic existence is understood as independent from epistemic approaches to it.

This differentiation between ontic existence and the epistemic approach guides the understanding of the measurement process and outcome (Table 1). A short look at a classical definition reveals a typical imprecision concerning the philosophy of science perspectives: All students of psychology learn a variation of this definition: “Measurement—a central epistemic activity in science—relates a number and a quantity in an effort to estimate the magnitude of that quantity” (Trout, 2001, p. 265). However, Trout continues: “A quantity is typically a property of a physical configuration, such as length or weight, and determines a function that applies to a domain or class of objects. At this high level of abstraction, the description of the purpose and relation of measurement is metaphysically neutral, leaving *open* the question of whether the domain is observable (empirical) or unobservable (non-empirical)” (Trout, 2001, S. 265, *my emphasis*). Here, Trout discusses ‘ontological’ and ‘epistemological’ questions because they are posed within the reasoning about science (the suffix “-logy” indicates it is about the study of anything, see Table 2). If a philosopher of science discusses that an object has a property, this is an ‘ontological’ question about the ‘ontic’ state of something. Trout claims that this definition of measurement is metaphysically neutral and does not imply realism or any other perspective. Yet we have to assert that Trout’s description is *not* metaphysically neutral because here, only the *epistemological* question of whether the domain is observable or unobservable is still ‘open.’ The *ontological* question of assuming that an entity has a pre-existing property is answered by this definition, therefore not an open question, and this reveals a classic realist philosophy of science position—that is, entity realism. Appropriate to the aims and logic of doing research in contemporary, canonical ways, psychological measuring is commonly understood as the ‘epistemic activity’ (see Trout above) of trying to arrive at an ‘as correct as possible truth’ about a quantity. Moreover, if researchers were to detect ‘problems’ with their measurement process, they would engage in overcoming these ‘problems’ and gaining a ‘better measurement process’, meaning that the outcomes represent ‘more correctly’ the true nature of the measured entity.

I conclude that most quantitative psychologists today still follow a strict entity realism ontologically speaking and that they understand the measurement process (Table 1) as an epistemic endeavor. They assume that their research objects have factual properties independent of any onlooker and that approaches to gain knowledge of these properties are more or less suitable to arrive at an as correct as possible representation of the factual property. They might be differently optimistic about what we can ‘learn’ about a property, a system’s state, its components, and perhaps the future, but always for epistemical reasons. The research objects are understood as having their nature, shape, quality, or property *per se*, and the task of science is to measure these as correctly as possible.

### 2.3 Responsibility of the researcher

Within the reasoning and as a logical consequence of this philosophy of science perspective—involving entity realism and the assumed difference between ontic existence and epistemic approach—scientists are not responsible for the characteristics of the research object. Researchers assume they only ‘discover’ what is already ‘out there’; they do not think they create entities—otherwise, it would be ‘flawed’ science. In this reasoning, researchers must ensure that the epistemic approach approximates the pre-existing entity as much as possible and as unbiased as possible. The conventional responsibility of researchers includes doing science as ‘objectively’ (Table 1) and ‘neutrally’ as possible to find the real characteristics of the investigated property.^2^

Why it is a crisis moment when replications fail is self-explanatory. Researchers hope they have found representations of real entities, relationships, and influences that are as correct as possible. The ability to replicate experiments means support for the claim that one has discovered an objective representation. Ideally, the results can also be measured by anybody else. If researchers cannot replicate a finding, it suggests that the previous finding was wrong. In section 4, I will argue that we will have a different view on replications and some different ideas of their ‘problems’ and ‘cures’ if we apply Barad’s agential realism for quantitative psychological science.

### 2.4 Full causality and determinism

The understanding of causality (Table 1) is that a vector of influence is transported from one process, state, or object to another process, state, or object. A cause generates an effect on another entity or process. The philosophy of science perspective of quantitative psychology is built on this understanding. It is assumed that causal processes happen in the world that researchers investigate, and causal processes are used to discover real-world processes. So, specific determinants are assumed to transport vectors of influence to occurrences like “racial bias” and, for instance, generate or modify it (like ‘empathy reduces racial bias’). For the discovery of real-world processes, the basic idea of an experiment is that different behavior or experiences between different conditions can be attributed to the differences between the conditions as their causes.

Determinism is the idea that all events are causally determined and that every outcome has at least one cause. As known, a deterministic system is characterized by the fact that previously existing causes unambiguously and completely determine its state in the future. Within determinism it holds that: If we find any variance empirically, there must be causes for this variance. If we cannot explain a variance, it is always attributed to epistemic reasons: we do not know enough about the determinants that cause the variance. Total determinism does entail that, ontically, there are causes for every outcome. Now, philosopher of psychology Gadenne states that strict and total determinism is not tenable for psychology; however, Gadenne argues that this is because of the *inexplicability* of chaotic processes and not due to indeterministic processes. The statement “chaotic processes follow strict causal laws, but are bounded by explicability and predictability” (Gadenne, 2004, p.125, my translation) exemplifies that Gadenne’s reason to question determinism as tenable is only epistemic—i.e., not being able to *know* about all determining influences. Gadenne does not assume indeterminacy ontologically. I argue that the research logic of quantitative psychology is built on total determinism, and all variance is attributed to epistemic issues. Even the unsystematic variance of every measurement outcome is understood as part of a so-called measurement error. Likewise, the attempt to gain more and more objectivity resembles the understanding that, in principle, there is a cause for every variance and that we are ‘bounded only by predictability’ (see Gadenne above).

## 3 Agential realism as the philosophy of science perspective for quantitative psychology

This section introduces Barad’s agential realism for the field of psychology. Barad was trained as a theoretical physicist and presents the alternative philosophy of science perspective with reference to physical objects, measurement processes, measurement outcomes, causal linkages, etc. This vocabulary makes the reasoning somewhat accessible for quantitative psychologists. Like them, Barad is talking about experiments. However, the agential realism perspective entails fundamentally different conceptualizations of science’s objects, processes, and outcomes (see some comparisons in Table 1 and more in detail explained hereafter).

“Knowledge’s are not innocent representations, but intra-actions of natures-cultures: knowledge is about meeting the universe halfway” (Barad, 1996, p. 189).

Barad negates the idea that with science, we find representations of real objects. Instead, Barad approaches realism concerning *entangled phenomena*. Before I enter the clarification of specific concepts, I look at the name ‘agential realism.’ Barad chose the term ‘realism’ because their aim is still to approach the ‘nature of nature’ or ‘nature of reality.’ The target is explicitly not “a matter of human experience or human understandings of the world” (Barad, 2007, p. 160). Barad chose the term ‘agential’ because this ‘nature of reality’ is understood as co-constructed by agencies (which need not be human). The underlying reasoning should become apparent after describing Barad’s framework and its possible application within quantitative psychology.

Barad draws heavily on the ‘philosophy-physics’ from Niels Bohr (although departing from it in specific issues). Barad examines at length the arguments between Bohr, Heisenberg, Einstein, and some of their colleagues in the 1920s and 1930s about some physical experiments. The arguments led to the famous Copenhagen interpretation of quantum phenomena, for which both Bohr and Heisenberg are held responsible but which is not in focus here. Despite their commonalities, there is a specific difference between Bohr’s and Heisenberg’s understanding of the ‘nature of nature’, which Barad draws upon. Both agree upon “the final failure of causality” (Heisenberg, 1927, p. 83), and they agree on this point: “what is wrong in the sharp formulation of the law of causality, ‘When we know the present precisely, we can predict the future,’ is not the conclusion but the assumption. Even in principle we cannot know the present in all detail” (Heisenberg, 1927, p. 83). The critical difference between the conceptions of Bohr and Heisenberg is the *reason why we cannot know* (the present) in all detail. Barad carves out that Heisenberg attributes the source that we cannot know in all detail to *epistemic reasons,* while Bohr attributes the source to *ontic reasons*. According to Barad, Heisenberg refers to a disturbance in the measurement process and centers on ‘possibilities of measurement.’ This disturbance in the measurement process is an epistemic question. Bohr, by contrast, centers on ‘possibilities of definition’ as an ontic question (see Barad, 2007, p. 301). Barad follows Bohr and assumes a certain *indeterminism at the fundamental ontic level of existence*. This is the first peculiarity of agential realism. Within this framework, the uncertainty ‘not to know in all detail’ is *not* due to epistemic problems but is indeterminacy at an ontic level.^3^ Importantly, this indeterminacy can be resolved. After a resolution, there are determinate states in the present, but they are also contingent on the (experimental) configurations. This is the second peculiarity of agential realism. A bounded object does not exist *per se* but is an outcome of a process; larger configurations resolve the indeterminacy into a determinate state. As a principle, these larger configurations belong to the outcome. These points and some corollaries are explained further in the following sections.

### 3.1 No entity realism, but realism toward phenomena

Agential realism does not assume individual objects with determinate boundaries or properties with determinate meanings as pre-existing *but as outcomes of processes*. By definition, this is no entity realism. Instead, reality “is composed not of things-in-themselves or things-behind-phenomena but of things-in-phenomena” (Barad, 2007, p. 140). This is to assume ontologically a thoroughly relational existence of everything; nothing exists independently by itself: “there are no independent relata, only relata-within-relations” (Barad, 2007, p. 429, footnote 14). In philosophy of science, the term ‘relatum’ (from Latin) refers to the object to which a relation proceeds; the plural form is ‘relata.’ In the examples ‘I see a particle’ and ‘I see empathy’, particle and empathy are each a relatum. Barad wants to express that there is no relatum without relations. To apply agential realism in psychology, I use ‘entity’, ‘object’, and ‘relatum’ interchangeably (see Table 1). The point here is, that none exists without relations. This builds on Bohr’s insight that, on the ontic level, the ‘nature of nature’ exists only in relation to specifics of (experimental) configurations. The reason that we can still deal with present individual objects is an occurrence that Barad calls *intra-action* (Table 1). Barad chooses this neologism (in contrast to ‘interaction’, Table 1) to express that intra-acting relata are not separate entities that influence each other or that one entity influences the other. Relata, objects, and entities do not preexist relations. As pre-assumption about the world, we should not assume any object—physical particles in the same way as psychological research objects like empathy—as existing without relations. The whole—a relatum and its relations—emerges only through specific intra-actions. Therefore, intra-actions enact ‘agential separability.’ What we see as boundaries between two seemingly separated relata, objects, or entities are *agential cuts* (Table 1). Barad uses the adjective ‘agential’ to express that the correspondent referent—‘realism’, ‘separability’, or ‘cut’—does not exist *per se* but that an agency brings this correspondent referent into being. The separability does not exist by itself but is agentially enacted. Intra-actions enact an agential cut but are themselves ‘agential.’ Importantly, there is no inevitable ‘agent’ behind the agential becoming. Humans can be agents but are not required. The term agential is just a marker for the understanding that a distinction between separate entities is an *effect* “in contrast to the more familiar Cartesian cut which takes this distinction for granted” (Barad, 2007, p. 140).

This fundamental relationality also applies to psychological research objects and entities such as Dienes (2008) examples ‘thoughts, images, networks, social pressures, social identities.’ Certain relations are crucial for the very existence of any relatum, and if these crucial relations are different, then the relatum is different. The relationality also applies when psychologists state that a concept like ‘social pressure’ is just like a molecule composed of much smaller atomic parts like ‘self’, ‘others’, ‘social norm’, ‘observable behavior in relation to that norm’, etc. That is, we should not think of any component as a distinct entity. Even if psychologists try to differentiate an occurrence like ‘social pressure’ into its assumed parts, according to Barad, no part exists without enacting relations. Relata and their relations are a conglomerate. That which is understood as an entity from the contemporary, canonical perspective is understood as an entity-within-phenomena or thing-within-phenomena (Table 1) from the agential realist perspective.

Phenomenon (Table 1) is Barad’s term for such a conglomerate. It is the name for the conglomerated relations. “It is through specific agential intra-actions that the boundaries and properties of the components of phenomena become determinate and that particular concepts (…) become meaningful” (Barad, 2007, p. 139). That means two entities might interact—as situated (!) separate entities—but their separateness is due to the encompassing phenomenon. The other way around is a phenomenon “produced through complex agential intra-actions of multiple material-discursive practices” (Barad, 2007, p. 206).

Instead of presuming entity realism, agential realism takes *phenomena* as the primary ontic unit of reality. This perspective recognizes a ‘reality’ (Table 1) in the sense of a shared experience. That is, discrimination can be ‘real’ (Table 1) for specific people and not for others. A physical object can be ‘real’ for specific radiation and not for others. However, this shared experience is presumed to be always bound to situations. This way, agential realism assumes we can encounter a situated reality, but composed of ‘real phenomena’ rather than composed of ‘real entities.’ Necessarily, a situated reality is bound to time and place. The term phenomenon refers to the relationality of each occurrence. Phenomena include the specifics of (experimental) practices and all these relations that are part of what seems to be a ‘real object’ in situations. Phenomena encompass the entanglements that enact a relatum-within-relations. Every noun we use in psychology, every psychological research object—for example, ‘thought’, ‘social pressure’, ‘sensibility’, ‘attitude’, ‘self’, and ‘stereotype’—must be understood as object-within-phenomena. To be more precise, we could call something an ‘object-within-phenomena’ when the contemporary perspective understands it as the smallest part and as the component of an occurrence. Likewise, we could call something a ‘phenomenon’ when the contemporary perspective understands it as an occurrence that is composed of smaller parts. However, I do not promote such a differentiation. After all, from the agential realist perspective, we do not need it because everything is entangled. For example, Gemignani et al. (2023) applied this agential realist perspective by understanding ‘migrants,’ ‘feminists,’ ‘oppressed,’ or ‘social justice’ as such phenomena.

### 3.2 Entanglement of ontic existence and epistemic approach

Understanding everything that we name with nouns as relata-within-relations—respectively as ‘phenomena’—and acknowledging indeterminacy until intra-actions place agential cuts implies that epistemic approaches cannot only discover what is already out there. Barad builds on Bohr’s insight that the configurations of an experimental apparatus (Table 1) co-create the outcome. These configurations are the compositions of material and discursive settings that enact certain intra-actions, not others. In physics, this can mean more, but not exclusively, material than discursive configurations. However, not surprisingly, there is no clear differentiation between material and discursive within agential realism. Barad explains an example of a cigar being necessary for the outcome of a specific physics experiment. However: “Not any old cigar will do: the high sulfur content of a cheap cigar is crucial. Class, nationalism, gender, and the politics of nationalism, among other variables, are all part of this apparatus” (Barad, 2007, p. 165). That is, the social category of gender is entangled with an ‘object’ like a cigar. Class is entangled with the necessary high sulfur content, and so on. The agential realist perspective sees these variables not as separate influencing forces but as parts of the apparatus. Accordingly, specific experimental apparatuses (Table 1) with their specific material-discursive configurations enact specific agential cuts. Likewise, epistemic approaches—in which researchers use specific apparatuses—enact intra-actions that set agential cuts.

A psychological apparatus (i.e., a psychological ‘instrument’), such as a questionnaire, has its own material-discursive configurations. Configurations of the apparatus itself—for example, the wording of questions—and configurations that enabled this apparatus beforehand. For example, we find historical and social changes in attitudes embedded in the logic of specific questions within questionnaires. Similarly, material phenomena, like the availability of telephones or the internet, at specific historical periods are embedded in the changes in attitudes and so on. This way, the apparatus itself is not a bounded entity. It is entangled in material-discursive configurations. Of course, every part of the apparatus is. There is no part, component, entity, or object of the apparatus not entangled with material-discursive configurations. Likewise, every part of an apparatus—like the wording of questions—is itself a specific configuration that enables specific agential cuts. When an agential cut is placed, this is the moment where the ontic indeterminacy is resolved into a situated reality. That means apparatuses are productive. In physics, they can materialize an object. In psychology, we might prefer to say ‘realize an occurrence.’ As said, this occurrence can be any research object or anything we name with words. Of course, this realization process is not restricted to experimental settings but is part of the ongoing dynamic reconfiguring of the world. For example, a specific questionnaire will—as apparatus—realize outcomes in an experiment, and a specific wording in cultural stereotypes will—as material-discursive configurations—realize occurrences in schools. Outside of (laboratory) experiments, Barad’s term ‘material-discursive configurations’ might be more suitable than ‘apparatuses’; for what they do, they can be used interchangeably. The important effect is that “apparatuses are not mere observing instruments but boundary-drawing practices” (Barad, 2007, p. 140). So are material-discursive configurations. If these configurations are co-creating what exists situatedly, then the ontic existence is not independent of the epistemic approach. Every epistemic approach establishes specific configurations and not others.

Agential realism assumes an inextricable entanglement of ontic existence and epistemological approaches. Then, by definition, a measurement outcome (Table 1) cannot be an ‘innocent representation’ of an independent truth (see section 3). If our apparatuses participate in realizing outcomes, then the measurement process (Table 1) is partly a creation. That is, to measure is not only an epistemic activity. To measure is an intra-action which leaves boundaries. To measure carves out one of the several possibilities. “The point is that measurement resolves the indeterminacy” (Barad, 2007, p. 280).^4^ The larger configurations, the material-discursive practices ‘take’ a measurement and produce agential cuts. To try to achieve independence of preferably every influence, as quantitative psychologists mostly do, is a fundamentally different approach than to assume that nothing exists without relations. In the agential realist perspective, we can never distance ourselves from that with which we intra-act. Importantly, we can never distance ourselves—not for epistemic reasons alone but because ontic existence is entangled with the epistemic approaches to it. This entails consequences for criteria of quality for science, which will be addressed in section 5.

“Practices of knowing and being are not isolable; they are mutually implicated. We do not obtain knowledge by standing outside the world; we know because we are *of* the world. We are part of the world in its differential becoming” (Barad, 2007, p. 185). Because one cannot disentangle knowing (Table 1) from being or epistemology from ontology, Barad again uses a neologism to explicate this point: “Onto-epistem-ology—the study of practices of knowing in being—is probably a better way to think about the kind of understandings that we need to come to terms with how specific intra-actions matter” (Barad, 2007, p. 185). Barad encourages us to understand the processes of emergence and not resign in the face of this entanglement. However, we need to learn onto-epistemology instead of continuing to try to approach an object ‘as neutrally as we can’, because neutrality does not exist—onto-epistemically.

There is also the issue of researchers’ placing agential cuts and, therefore, taking part in the world’s differential becoming. This impact goes beyond the handling of research outcomes (like the handling of outcomes discussed in the realm of the atomic bomb) but operates at the level of co-creating the research outcome itself. This is why Barad also demands to imply ethics: “[W]hat we need is something like an ethico-onto-epistem-ology—an appreciation of the intertwining of ethics, knowing, and being” (Barad, 2007, p. 185). Notably, the concrete ethical line cannot strictly be derived out of agential realism, only that we should fundamentally imply ethical considerations because we cannot purport that we only discover what is out there but take part in the formation of what we ‘find’ or ‘create’, respectively. This implicates the responsibility that researchers have.

### 3.3 Responsibility of the researcher in agential realism

Within agential realism, researchers are not only responsible for the kind of knowledge that they seek “but, in part, for what exists” (Barad, 2007, p. 207). To the degree that human practices are involved in the intra-active becoming of the world, humans are agentive participants and co-creators of the world. Notably, within agential realism, this boundary-drawing is not an unwanted influence that must be eliminated but an inevitable part of the phenomenon. If researchers agentially set boundary-drawing apparatuses, they have to question what exactly they ‘measure.’ If measuring is carving out one of several possibilities, researchers chose one particular possibility. It became famous that Isaac Asimov (1920–1992) doubted if ‘intelligence’ is just that, what the ‘intelligence test’ measures. Within agential realism, psychologists must doubt if constructs are indeed ‘just that, what the test measures.’ We cannot conduct any psychological experiment, exploration, or analysis without using a particular line of thought, specific language, certain nouns and verbs, maybe pictures, graphs, or icons. All these, too, are embedded in their social-material-historical entanglements. They have specific meanings for certain people in certain constellations at certain times and places and other meanings in others. Accordingly, researchers’ decisions about design and material, along with all their histories and entanglements, are also part of the boundary-drawing practices in research settings.

These decisions of researchers can have strong and far-reaching consequences. For instance, Teo (2008, 2010) refers to Spivak (1988)—who coined the term ‘epistemic violence’—and transferred this as ‘epistemological violence’ to psychology to stress that this violence is executed in knowledge production. Teo concentrated on situations where interpretations of data “implicitly or explicitly construct the *other* as inferior or problematic, despite the fact that alternative interpretations, equally viable based on the data, are available” (Teo, 2010, p. 298, emphasis in the original). One could expand this logic and name it ‘epistemological violence’ whenever researchers’ decisions within study designs create negative consequences for some people. It is this important shift in the idea of research that researchers are partly responsible for what exists. Then, researchers have to include ethical considerations, such as developing criteria for judging what a ‘negative consequence for people’ is, in order to derive rationales for decisions about research design and material.

At the same time, it is important to state that researchers do not have full control over an outcome: “not everything is possible at every moment” (Barad, 2007, p. 182). Researchers do not have the opportunity to do every intra-action they might want. They are themselves just a (relational) part of the material-discursive practices. They are researchers-in-relations and use language-in-relations and experimental designs-in-relations with groups-in-relations and so on. With all their agentive practices, researchers are neither fully responsible nor not responsible for co-creating the outcome. Does this allow us to ask how big the researchers’ ‘part’ is? If there are either previous or non-researcher agential cuts, could we then not ask: where is the line between researchers’ responsibility and their non-responsibility? Such a question might arise from the hope of being able to distinguish between situations where experimental design decisions have an impact on the shape of the outcome and situations where they have only too little or no impact. It is the idea that configurations other than the researchers’ have set most of the agential cuts, and the researchers’ possibilities for influence are negligible. Suppose there are, in principle, phenomena where researchers cannot sufficiently co-construct the outcome. In that case, it seems reasonable to try to distinguish such phenomena from others where the construct is just that, what the test measures (see beginning of section 3.3). However, the notion of an ‘extent of influence’ resembles the conventional idea of a possible separation between a phenomenon and the influencing configurations or between the ‘humanly discursive’ and the ‘non-humanly material’ practices. Instead, Barad states: “Indeed, it is through such [material-discursive] practices that the differential boundaries between humans and nonhumans, culture and nature, science and the social, are constituted” (Barad, 2007, p. 140). Any separation we find as an outcome is an “agential separability—an agentially enacted ontological separability within the phenomenon” (Barad, 2007, S. 175). This means the separation is not pre-existing and just there to be found, but different configurations will enable different possibilities for agential separation. This leaves us with a reasonable desire to disentangle different influences because we have the hope to decide where researchers can do ‘better.’ At the same time, we have to acknowledge that such a differentiation is onto-epistemical—and onto-epistemological (see Table 2)—impossible because of the inextricable entanglement of material-discursive practices. Ways of implementing this understanding in agential realist psychology are discussed in section 4.3.

### 3.4 Reworked causality and emerging possibilities

Incorporating indeterminacy and fundamental entanglement in a thinking model does not mean there is no causality. However, this means reworking the previous canonical understanding of causality (Table 1), which is about the ‘relationship between distinct sequential events.’ Agential realism rethinks this in terms of intra-activity: “Intra-actions do not simply transmit a vector of influence among separate events. It is through specific intra-actions that a causal structure is enacted. Intra-actions affect what’s real and what’s possible, as some things come to matter and others are excluded, as possibilities are opened up and others are foreclosed” (Barad, 2007, p. 393). This way, we are invited to think of causality as entangled with conditionality. Causes also exist, but not as a sole reason but only together with conditions that render a causal chain possible (and then any given outcome is not the only possible). There is a causal impact in intra-actions but not as the ‘transmit of a vector of influence among separate events.’ It is sort of a thinking of neither ‘anything goes’ nor ‘total determinism’ when Barad talks of the “open-ended becoming of the world which resists acausality as much as determinism” (Barad, 2007, p. 182). In Barad’s ongoing becoming, we find both the renunciation of then-separate entities and the implementation of context, conditions, and configurations that enable a causal execution.

This perspective encompasses both the confinement of possibilities and the multiplication of possibilities. “Intra-actions reconfigure the possibilities for change. In fact, intra-actions not only reconfigure spacetimematter but reconfigure what is possible” (Barad, 2007, p. 182). On the one hand, possibilities for an outcome are confined by intra-actions that set certain agential cuts. Confronted with such an agential separability, researchers cannot realize any outcome they might wish. This acknowledges that variables-in-relations can impact other variables-in-relations, and sometimes we cannot escape some impact. Quantitative psychologists are used to the idea that they only observe the interaction of variables. However, compared to a deterministic understanding, agential realism assumes the existence of several possibilities for an outcome. These possibilities (a) have partly no further reason because there is some fundamental indeterminacy, and (b) have partly the intra-actions as reasons, which can possibly be realized differently. That means there are two sources for the multiplication of possibilities for an outcome. While determinism holds that there are always reasons for a specific outcome, agential realism opens the question of where the world can be realized differently. The different consequences of applying issues (a) and (b) in quantitative psychology are enfolded in sections 4.1 and 4.2.

Agential realism also reworks the understanding of what is ‘objective’ (Table 1): “[O]bjectivity in an agential realist sense requires a full accounting of the larger material arrangement (i.e., the full set of practices) that is part of the phenomenon investigated or produced. (To do otherwise is to misidentify the objective referent). Hence objectivity requires an accounting of the constitutive practices in the fullness of their materialities, including the enactment of boundaries and exclusions, the production of phenomena in their sedimenting historiality, and the ongoing reconfiguring of the space of possibilities for future enactments” (Barad, 2007, p. 390–391). Objectivity, then, is about communicating the larger configurations of a boundary-drawing apparatus. Put simply, if we manage to inform colleagues about most of the involved relations of the investigated relata-within-relations, then we increase the possibility that they can reproduce these involved material-discursive practices and the realization potential of this phenomenon. Agential realist objectivity is not about eliminating influences but about communicating material-discursive practices as much as possible.

## 4 Consequences of applying agential realism in quantitative psychology

Barad proposes a meta-theoretical perspective, which I discuss as a philosophy of science perspective suitable for quantitative psychology. Many issues highlighted through the confrontation with agential realism have already been discussed (sometimes extensively). For example, to question the inherent boundaries of research objects and the necessity to take producing configurations into account has already been (and for a long time) discussed by Gergen and Gergen (1988, 2003), who propose that the self and every psychological construct are relationships rather than individual entities. K. Gergen implements this understanding in everyday life and professional practices, such as education, therapy, and knowledge production (Gergen, 2009). So, I propose agential realism not because it would offer completely new conceptualizations. However, it offers a set of bundled assumptions and corollaries that can be pretty accessible for researchers trained in quantitative logic because both approaches discuss experiments, objectivity, apparatuses, measurement, or knowledge acquisition (see also Table 1).

The pre-assumptions described in section 3 have several consequences for quantitative psychology, but here I focus on four: 1. The agential realist perspective changes the conception of a ‘true score.’ 2. It changes the conception of the context. 3. It changes the conception of the researchers’ responsibility. 4. It changes the conception of the research endeavor.

### 4.1 Indeterminacy means there is no true score

The first issue arising from Barad’s reasoning is the idea that there is a core indeterminacy in our world. This, however, is not an uncertainty due to the epistemic reason of ‘not knowing well enough’ but due to the ontic reason of ‘not being determinate’ at a certain level of existence. We can imagine that—in *certain situations*—this indeterminacy can be too small to have a relevant impact. However, which situations are concerned can be treated as an empirical question and cannot be a pre-assumptions. Further, the question of whether an impact is ‘relevant’ will again depend on the context and aim of the research. The agential realist pre-assumption is that a certain indeterminacy is part of the phenomenon a researcher is interested in. This indeterminacy is the reason for an unexplained variance.

Let us imagine repeated measurements under consistent conditions, at least in theory (because they are hardly realized in psychology). If we repeat any measurement, we will achieve varying values even if we do not change conditions. These variations are termed variance; alternatively, we utilize the square root of the variance, known as the ‘standard deviation.’ Traditionally, this variance is perceived as comprising both ‘systematic’ and ‘unsystematic error.’ The first is perceived as stemming from an unwanted influence, which has to be eliminated to approach the ‘true’ unbiased score. For the ‘unsystematic variance’, it is acknowledged that it cannot be eliminated, but it is conceived as indicating where the assumed ‘true score’ may lie. In this way, the traditional conception treats the unsystematic variance as stemming from pre-assumptions problems like ‘not knowing well enough’ where the ‘true score’ may lie. Consequently, the distribution curve of this variance is then used to infer the assumed ‘true score’ while admitting a little ‘uncertainty.’ However, from the agential realist perspective, this unsystematic variance stems from ontic indeterminacy. Consequently, there is no assumed single true score behind a blurred measurement process but a variance of possibilities. We can think of the distribution curve as showing the probability of each outcome but with the alteration of assuming an indeterminacy within a certain range instead of uncertainty about one true score. With an agential realist perspective, we have to assume a realization potential—i.e., the unsystematic variance distribution curve—for each specific configuration setting instead of one true score.

#### 4.1.1 Every system has a realization potential

Whenever we psychologically ‘test a person’, we have to assume that there is an inherent variance within the investigated ‘feature’, which is actually a ‘feature-system.’ The term ‘system’ is added here to indicate that agential realism does not assume features that can be measured but that a feature is carved out of a larger phenomenon through material-discursive practices (including measuring devices). That we have to assume an inherent variance applies even if we could repeat the larger configurations of the situation exactly. This variance is not due to a measurement error but due to an inherent part of the whole phenomenon. When we obtain a particular realization, the variance is only broken down to a particular outcome value because of intra-actions, which cause this realization out of the larger potential of realizations. If we were able to repeat the same intra-actions, we would nevertheless obtain a more or less different result. Within the realization potential, this more or less different result has no further reason to be different but is only more or less likely. Hence, ‘measuring’ a ‘feature-system of a single person’ means obtaining information about this specific system’s realization potential within the given configurations. Hence, researchers no longer search for an assumed single true score but for a range of realization possibilities.

#### 4.1.2 Consequences for comparisons

Importantly, this conceptualization changes the comparison between people. Researchers then do not compare two different scores—no matter whether true or estimated—but we compare two distribution curves. Whenever these curves overlap, this brings similarities instead of differences to the fore. When comparing such potentials between persons, it is quite possible that in specific configurations and concerning a specific scale, person A shows a realization potential different from person B: different concerning the mean and/or different concerning the standard deviation and/or kurtosis of the realization potential. Whereas in the conventional understanding, the curves were used to deduce a significant difference, this agential realist conceptualization highlights the overlap of the two distribution curves. Within the overlap a difference as well as no difference can *occur with no further reason* other than the ontical indeterminate variance. If the realization potentials of two feature-systems overlap, then in agential realism, researchers do not consider this as an ‘inner difference’ but as ‘a sometimes realized difference and a sometimes realized sameness.’

#### 4.1.3 Consequences for replication from indeterminacy

The first critical consequence for replicability is that, from an agential realist perspective, the replication rate has an onto-epistemic limit. A replication rate of 100% is, in principle, not possible for onto-epistemic reasons. It is not that theoretically 100% was the ideal that we cannot reach for epistemic or practical reasons, but that incorporating a fundamental ontical indeterminacy limits the possible replication rate for each phenomenon. The rethinking of ‘true scores’ in the form of realization potentials has a critical impact. Realization potentials increase the overall variability of results for any setting of conditions. Suppose we have to aggregate data of a group of people. In that case, we do not assume the property as having a determinate value somehow ‘inside the person.’ Instead, we assume kinds of ‘individual phenomena’—with all their entanglements—which we aggregate. The idea is, in some situations, it might be possible to realize a specific property, but in principle, this happens in the form of a specific variance. Let us continue to theoretically assume that we were able to repeat the same configurations. If we incorporate the indeterminate variance of each ‘individual phenomenon’ and analyze a group of people, this understanding increases the ontically caused variance of the group. This is because every individual brings more than only one value to the group’s variances.

Concerning the replication of studies, I argue that until now, psychologists have taken an outcome as indicating an assumed true score instead of one of the possibilities. This is relevant when comparing two outcomes, for instance, from an older study and a replication study. A replication of psychological studies often tries to replicate a significant difference between two groups. The replication fails if the significant difference was shown in a previous study but not in a recent study. Suppose we assume realization potentials instead of single true scores. In that case, it might well be possible that both outcomes, the difference outcome and the no difference outcome, are part of their regular group-comparison realization variance. Imagine we could estimate each group’s whole regular realization potential, and both curves might overlap partly. If we realize an outcome of each group in one study, in most cases—unless the distributions hardly overlap, but even in cases where the distributions totally overlap—this applies: Finding a significant difference has one particular possibility, and finding no significant difference has another particular possibility. Importantly, in the agential realist perspective, this stems from ontic reasons and not from epistemic ones. To find a difference or not with specific possibilities belongs to the phenomenon and is not a measurement error. We are neither supposed to find a significant difference in each comparison nor to find no difference. The realization of a difference and the realization of no difference can be part of the configurated possibilities. Accordingly, if we (at least theoretically) replicate the same configurations many times, we could look at a proportion of the realization of differences and a proportion of the realization of sameness. By that, we judge a replication study’s outcome differently than up to the present. It is mostly no longer about whether mechanism A exists or not. It then is about the question of how often—out of the ontic possibilities—mechanism A might realize (within these configurations) or not.

Of course, other problems related to a low replication rate—like publication bias, flawed research, or false positives—still exist. However, from the agential realist perspective, another issue is added: A low replication rate of a specific mechanism does not necessarily prove that phenomenon P does not exist, but it can prove the regular indeterminate variance of this phenomenon, that, for example, sometimes results in a group difference and sometimes not. Researchers have to discuss the basic idea of replications and the meaning of replication study outcomes anew when applying the agential realist perspective.

### 4.2 Configurations as part of things-in-phenomena

A second important alteration in thinking arises from Barad’s reasoning that the relations are always already part of the relata. These ralata-within-relations only exist due to configurations (or larger apparatuses) that set agential cuts via intra-actions. Agential realism assumes that the objects and outcomes we find are materializations/realizations of material-discursive practices. Outcomes are sedimentation of intra-actions of these practices, which cause agential cuts. This framework states there is no such thing as ‘ingroup-favoritism’ or a ‘representation of the other’ in our mind without larger configurations co-creating it and indeed being part of what we named ‘favoritism’, ‘representation’, or any other construct. There is no thinking, feeling, or behavior without material-discursive practices that set agential cuts around a then-named thought, feeling, or behavior. This is a fundamental shift toward a relational ontology. Not a single psychic phenomenon exists without its history of entanglements and ongoing material-discursive intra-actions enacting agential cuts. Nevertheless, we should not misunderstand Barad’s phenomena as deterministic systems and should not repeat the search for determined causal chains within them. As mentioned, even causality is something enacted through specific intra-actions. With that in mind, we cannot treat context as a third influencing variable.

#### 4.2.1 Context is not a third variable

The agential realist perspective also implies an alteration of psychologists’ understanding of their objects ‘in context’: “The notion that human psychology [psyche]^5^ is shaped by the social context has been the central premise of the field for nearly a century” (Van Bavel et al., 2016b, p. E4935). However, the idea that the context ‘transmits a vector of influence’ toward human cognition fundamentally differs from Barad’s notion of intra-actively enacted agential cuts and the ongoing becoming of the world. Within agential realism, cognition is *not a relatum that is influenced by* its separate-from-the-object surrounding context. Rather, any cognition, feeling, or experience is a material-discursive phenomenon contingent on historical and actual configurations. Also, what psychologists understand as basic, universally human, not-social, or enduring is a contingent outcome of larger configurations. ‘To be shaped by’ is exactly *not* what Barad understands of relata-within-relations. Van Bavel and colleagues assume a classic causal influence, whereas Barad assumes a co-creation. Within agential realism, we can understand the human psyche and all its contents as entangled in material-discursive practices, and the objects of psychological (!) interest, as well as the psychic states, are realized differently within different practices.

Concerning the demand to take the context as part of the phenomenon into account, there seems to be a growing willingness, for instance, within social psychology, to attach more importance to surrounding configurations. Both within the replication debate (e.g., van Bavel et al., 2016a) and as a principle (Weber et al., 2023), psychologists have promoted that we need to understand psychology’s objects as context-sensitive and context-embedded, respectively. Pettigrew even suggests celebrating: “Contextual social psychology is finally emerging” (Pettigrew, 2018, p. 969). Jost promotes a new journal that embraces the context-embeddedness of psychical^6^ phenomena because “we cannot stand outside of history—or culture or politics or economics” (Jost, 2024, p. 7). At first glance, this sounds like Barad’s request. However, I want to stress two important issues: *First*, it makes a difference whether we conceive of this influence as a moderator variable, like a ‘third’ variable that ‘transmits a vector of influence’ and determines how the effect of variable A to variable B unfolds, or as inextricably entangled part of the phenomenon. Cultural psychology has extensively discussed their latter perspective as different from the conception of ‘moderating.’ Cultural psychology, as, for example, Chakkarath (2011) and Chakkarath and Straub (2020) describe it, is based on the principle that culture and psyche evolve through a reciprocal, mutual co-construction. Psychic structures, processes, and functions are understood as inherently entangled with cultural lifestyles, practices, languages, and discourses and non-existent without their context.^7^ *Second*, and this point might be a consequence of the first one, psychologists often apply the embeddedness primarily to the cognition or emotions of the research participants and rarely to the researchers. For instance, Pettigrew does not transfer the insight that “cultures and social norms moderate basic psychological processes”^8^ (Pettigrew, 2018, p. 963) to the idea that researchers, their apparatuses, and the language and concepts they use are also always entangled within cultures and social norms—and what this entanglement means for the research process. Weber et al. (2023) acknowledge that “researchers are themselves embedded in systems of knowledge production,” but that, importantly, is their final sentence and not the basis of their reasoning.^9^ The most far-reaching application of the idea of embeddedness within social psychology seems to be undertaken by Cikara et al. (2022). They discuss various possible contexts/configurations, including “political, legal, research and regulatory institutions” (p. 545) as productive for social categories. They explicitly include the researcher’s responsibility and address “the authoritative power given to science to shape truth and knowledge” (Cikara et al., 2022, p. 537). I reckon that their recommendations about study design and analysis choices can be founded on agential realism. Even though some approaches to context-sensitivity do not go as far as agential realism, a future agential realist psychology can still learn from such perspectives, for instance, regarding the application of specific methods. Pettigrew (2018) and Jost (2024) advocate multilevel modeling to link different levels of complexity. Skinner-Dorkenoo et al. (2023) demonstrate a systemic approach to racism. A thorough examination of such methods in relation to the basic assumptions of agential realism is one of the future tasks for agential realist psychology, which I address in section 5.

#### 4.2.2 Consequences for replication from entanglements

As a consequence for replications, we must always consider the configurations of an outcome as part of our research questions and objects. We shift the focus to understanding an outcome beyond previously assumed inherent features (such as a person’s characteristics) to encompass instead the entire producing phenomenon, including configurations previously considered outside of the investigated feature. This includes historical, material, and researchers’ entanglements. To replicate ‘ingroup-favoritism’, for instance, we must consider the larger relations that render such an outcome possible. This often may require replicating those relations as well. Barad states: “Crucially, the objective referent of measured values is phenomena [*sic*], not (some abstract notion of) objects (which do have an independent existence)” (Barad, 2007, p. 340). We remember that psychological ‘objects’ need not be physical ones but can also be characteristics, etc. Accordingly, we must try to replicate situated phenomena using the reasoning described in section 4.1 rather than trying to replicate essential mechanisms or characteristics that are assumed to be universal.

For different reasons, psychologists have already discussed how neglecting the context can reduce the replication rate (e.g., van Bavel et al., 2016a), although discussions are about objections to context relevance and contingency circumstances. For instance, Landy et al. (2020) demonstrated the importance of operationalization choices for obtaining the same or at least similar results after conducting a study. Nosek et al. (2022) give a sophisticated overview, but they part from agential realism in important points. For one, they assume a total determinism, which can be read out of reasoning like “[An outcome reproducibility failure] can occur because of an error in either the original or the reproduction study” (Nosek et al., 2022, p. 721). This reasoning contradicts the inclusion of a fundamental indeterminacy explicated in section 4.1. Besides the mentioned onto-epistemical limit for a replication rate given existing indeterminacy, there are two more critical issues concerning the conceptualization of the context. The first is the necessity of considering the larger configurations. Nosek et al. (2022) promote caution against ‘unconsidered factors’ (p. 727), but they do not seem to see this necessity for every replication procedure. From the agential realist perspective, every finding is necessarily contingent on its configurations. Second, any ‘unconsidered factors’ and enabling conditions must be understood as outcomes-with-enabling-configurations in themselves. These factors are not variables with inherent characteristics or independent working processes that influence the primary object of interest. A ‘racial bias’ should not be understood as a feature of a person but as a culturally enacted phenomenon. It is a possibility within a culture system that is enabled through configurations. This cultural possibility has many more components-in-relations that must be accounted for. This accounting should not proceed deterministically, assuming bounded entities that have characteristics. It is not that ‘racial bias’ is a feature of the culture either. Features are not to be located within an ‘object’, no matter if the object is a person, a family, or a culture.

From an agential realist perspective, too much information is lost when researchers do not account for larger enacting configurations of a phenomenon. Furthermore, important information gets lost if researchers search for essential characteristics or ‘vectors transmitting an influence’ instead of co-creation. If the investigation is directed toward transmitting vectors, it misidentifies the investigated referent. As the first step, instead of eliminating ‘influences’, researchers must work *with* them. As the second step, researchers also need to search for enabling configurations in a nonessential way. This influences the idea of the research endeavor and touches on the question of generalizability. We can no longer think of realizations as widely valid as classic approaches assume, which presuppose that realizations exist independently and are merely biased by context. If a finding is a co-creation of relata-within-relations, then generalization is in question. I address this in section 4.4.

### 4.3 Tasks for responsible and accountable researchers

Section 3.3 clarified that researchers have a broader responsibility with an agential realist perspective than a classic approach. A new responsibility is added to previous responsibilities (like honest behavior, transparency, etc.) because researchers’ decisions may play a part in the phenomenon’s becoming. In this section, I discuss where to put some attention when we implement this understanding of the possible ontic involvement of researchers. If there is no underlying separation between the researcher’s influence and non-researcher configurations that can potentially be detected, then we will rarely try to find such a demarcation line and instead start to learn to deal with this entanglement. There can be co-creations from researchers’ decisions that cannot be eliminated because they are an inherent part of the phenomenon. That is why researchers cannot only rely on the strategy of trying to eliminate their part-taking. Part-taking must be fundamentally acknowledged and concerned with the following (amongst others).

#### 4.3.1 Decisions must be justified

One consequence of this alteration is that findings are not as widely valid as classic approaches assume, which presuppose that a characteristic of a research object is, in principle, independent of the researchers. In section 4.2, I already discussed the limitation of general validity due to the context relevance of each phenomenon. The outcomes’ dependence on researchers’ decisions is a further limitation. When researchers cannot declare that they only study what is already out there, then they have to declare why they are studying the phenomena in the way they do. Then, the question of how to design research is not only about operationalizing a research question in the best way to represent an assumed pre-existing characteristic but also about why researchers build and frame the parts as they do. Why do researchers use certain language, conceptualize something one and not another way, frame a question this way and not another way, etc.? When researchers acknowledge that they play a role in the research outcome, *every decision about a research design must be accounted for rather than being self-evident.*

#### 4.3.2 Ethics must be made explicit

Researchers need new criteria for accountability. Classic criteria for research quality do not suffice here because, within the perspective of entity realism, there is no need to justify the framing of a question beyond the examination of whether a design is an appropriate method to represent what is already there. Within agential realism by contrast, there is the possibility that a phenomenon could be realized differently, and researchers have to justify why they take part in a particular becoming and not in another one. This again makes clear why Barad proposes that we need an ethico-onto-epistemology. However, it is already clear that agential realism does not prescribe *which* ethical lines should be followed. Researchers have to explicate their ethics (and maybe a scientific community starts to discuss agreements about ethical lines in specific times and places).

From an agential realist perspective, we must start with situated guidelines instead of generalized ones. For the scope of this paper, we might orient toward rights like the right to life, liberty and security of the person, and freedom of thought, opinion, and expression. Such guidelines will imply striving to eliminate violent or discriminatory research outcomes. If researchers agree on such rights and an outcome still diminishes the freedom of expression of persons, then researchers are co-accountable.

Categorization into groups may be a prominent example of psychology’s part-taking in outcomes. From the conventional perspective, some research objects or persons supposedly possess common features that other objects/persons do not have (or to a significantly lesser extent). This is a common reason to categorize them into groups.^10^ Applying the agential realist perspective, we do not locate features within distinct objects, so this rationale for categorization is not applicable. A category does not present itself as self-evident. Instead, we always have to explain the rationale for grouping people in a certain way. This does not make categories useless; we can have good reasons for categorizations, but we have to tell those. However, it stresses the relativity of categorization and demonstrates the contextuality. Again, this clarifies that we need ethical explanations for categorizations and cannot disguise that there are choices behind our groupings. We have to confront researchers with the danger of executing epistemological violence (i.e., violence executed in knowledge production, see section 3.3) because researchers cannot return to the statement that they have just found what is there, independent from them. The same applies to the identification of differences. Differentiation could be a meaningful and appropriate action, but this, too, is contingent and a realization-within-relations. Again, it demonstrates the need for ethico-onto-epistemological considerations.

### 4.4 Altered research endeavor

Initially, I described psychology’s research (Table 1) endeavor as an attempt to describe, to understand, to explain, and, in some cases, to be able to change human thought, feeling, and behavior. This conventional research endeavor is about knowing (Table 1) the mechanisms that determine results. This way, researchers suppose that they can explain why things happened in the past and hope they can predict what will happen in the future. Kim (1999), in order to develop an alternative, described the contemporary, canonical research endeavor as an attempt to find the ‘periodic table of basic human behavior.’ The identified basic ‘elements’ could then be used to explain even complex human behavior. Additionally, researchers hope that knowing the mechanisms allows them to intervene and sometimes control an outcome, at least a little bit (e.g., to help somebody feel better). This reasoning is based on the deterministic idea that the system’s state at one point determines the state later. The agential realist perspective alters this reasoning in two critical ways: It includes an indeterminacy within causal processes. Moreover, it understands the components of any system as contingent from its enacting configurations (plus the indeterminacy also within this enacting processes), so that we must assume a connection of everything with others (i.e., relata-within-relations). No entity or process is disconnected, and no system within the universe is enclosed and separate from the rest.

These points change the research endeavor. The indeterminacy within processes diminishes the predictability on an ontic level. This understanding establishes variance as a regular part of every mechanism. Again, in the agential realist perspective, not everything is possible, but in each situation and configuration, more than one outcome is possible—for onto-epistemic reasons and not as epistemic fallacy. Furthermore, other configurations might disable specific realizations and enable new ones. This implies that we search for possibilities instead of the one true result. If researchers find one realization, a question arises about what other realizations might look like. Then, research is not only about ‘how it is’ but also about ‘how else can it be?’ The agential realist psychology accounts for possibilities. It disengages the idea of finding human mechanics that will repeatedly work the same way. Instead, psychological research (Table 1) is about psychic and behavioral possibilities. Agential realist psychological research strives to describe, understand, and explain the *possibilities* of human thought, feeling, and behavior—within specific configurations (including those of the researchers). That entails that research can look at specific realizations, the configurations of these realizations (including researchers’ configurations), and other possible realizations (and their configurations). This is an alteration of research questions.

#### 4.4.1 Altered research questions

Agential realism alters research questions. I will consider three types of research questions in the following three paragraphs. *First*, the formerly common question about ‘the character of X’ can still be pursued. However, any outcome is an answer about a *local and temporary phenomenon*, and extra attention needs to be given to ask for the scope of this contingent realization. *Second*, agential realism shifts our attention to the character of the enacting configurations and does not locate ‘the character of X’ only within a bounded entity. For each situated realization, researchers are simultaneously provoked to ask: What enabled this outcome? *Third*, agential realism directs researchers’ attention to what was disabled before and what else can be enacted. If relations render some relata possible and others not, we can investigate which other relata can be realized. Above all, researchers have to justify why they follow a specific research question, use a particular study design, and put a particular configuration of their research apparatus—nothing can be just a matter of course.

Concerning the *first* type of research questions, which is about investigating a local and temporary feature, we can note: “The line between subject and object is not fixed, but once a cut is made (i.e., a particular practice is being enacted), the identification is not arbitrary but in fact materially specified and determinant for a given practice” (Barad, 2007, p. 155). A relatum can become situated ‘real’, even though ‘being real’ is then not about being existent without an onlooker/interaction (see classic realism) but about situatedly shared experiences of intra-actions and cuts (see Table 1). Researchers can be interested in investigating a *situated property*, a *local quantity*, or a *temporary character* of an entity-within-relations. Especially so-called ‘applied research’ is used to deal with phenomena that might have an important, situational impact but are limited by their scope. In the same way, so-called basic research must develop an understanding of any investigation as research about realizations within *local and temporary* conditions.^11^ Such (onto-epistemological) knowledge can be very interesting for certain people and specific goals. However, a psychological study cannot reveal something about every human.

Concerning the *second* type of research questions, which is to investigate the enacting configurations, in some areas, new research questions might emerge. Instead of concentrating on the realizations that we find in our worlds, we can and should also ask what creates them and what brings them into the world. Especially if we want to use an outcome, for instance, ‘persons X react to Y with Z’, then we need to know more about the enacting material-discursive practices since we cannot assume the mechanism resides within people. The characteristics of anything are more sensibly located in relations than in entities. Researchers can no longer search for essences because they are not located in an entity but instead are an outcome of enabling configurations that researchers can investigate.

Concerning the *third* type of research questions, investigating what else can be enacted is whether other realizations can be carved out of the possibilities. This makes realizations less self-evident. It opens up the question of whether things could be otherwise and if realizations could be different. If the boundary-drawing apparatuses have specific configurations, we can research if and what other configurations can enable. This understanding also can generate whole new research questions. For every finding, we could start to ask, ‘Can it be different?’ This links to Barad’s reminder that ethics play a role in the researcher’s decisions because if ‘it can be different’, then we need to answer the question, ‘Which difference is desirable and why?’ Besides the new perspective on changed configurations, this alters the understanding of any first outcome as not a given but as *one* possible situation.

#### 4.4.2 Altered interpretations

The ethico-onto-epistemology of the agential realist perspective alters interpretations of outcomes. The alterations are implicitly mentioned in the discussion of altered research questions in the section above: Any outcome is interpreted as a local and temporary realization, and it is a different research question of how far it might spread. Any outcome is not interpreted as residing within a person or an entity but in material-discursive practices and configurations, enabling this outcome. Any outcome is interpreted as one possibility amongst others, and it is a different research question of how frequently different realizations emerge.

Transferred to social situations, this touches on numerous interpretations. For instance, that any outcome is interpreted as one possibility amongst others transfers an insight from ‘the human psyche saves energy through categorization’ to ‚the system-of-human-psyche has the capability to save energy through categorization.’ This changes the point of energy-saving from a ‘must’ to a possibility. Such an outcome—to save energy through categorization—is one possibility amongst others, depending on the configurations of the material-discursive practices plus an indeterminate variance (until intra-actions carve out one particular situated outcome). It changes our view on ‘psychic mechanisms’ when we no longer search for the hard-wired program in brains and minds but see possibilities within configurations. Then, we can ask for the situations and configurations when people do not categorize to save energy. This perspective opens up for the change of statements like ‘humans automatically perceive skin color and gender’ to ask ‘under which configurations do human-systems not perceive skin color and gender?’ Every given realization is not the only possible one.

Furthermore, looking for enabling configurations can change the interpretation of a locus of control. A ‘racial bias’ is then located not only within a specific person but also in the structures of society, the current language, thinking models, narratives, etc. A score in an implicit association test for racial bias is then interpreted as an indicator of cultural associations and not only individual ones.

When we see realizations as local and temporary, we cannot interpret them as elements of an assumed ‘periodic table of basic human behavior’ (as criticized by Kim, 1999). This changes the idea of generalization. From an agential realist perspective, generalizability is not a goal *per se*. Instead, we have to assume there are constellations of material-discursive practices that spread across every human on earth and constellations with a much smaller scope. It is an empirical question of which constellation realizes where and how often. Needing to breathe oxygen with lungs might be such an earth-wide (nowhere near ‘universal’) configuration for humans; needing to reduce cognitive dissonance (*cf.* Festinger, 1957) might not be earth-wide. Notably, the outcome that the need to reduce cognitive dissonance is *possibly* an earth-wide human phenomenon could be an empirical finding. However, I suppose these earth-wide configurations are rare for the psyche and psychology. Instead, with an agential realist perspective, we do not seek generalizability but understanding a specific situation, including its indetermined realization potential. Landy et al. (2020) organized 15 research teams to test the same research question, each with its own operationalization. They showed overall ‘how design choices shape research results’ to learn how to approach generalizability. However, with an agential realist perspective, a project like that would try to use the divergence of the results to learn something about the specificities of each operationalization. Not Generalizability is the goal *per se*, but knowledge about local and temporary phenomena.

## 5 Further directions

To take agential realism as the philosophy of science perspective for quantitative psychology changes assumptions about ethico-onto-epistemological basics, changes the procedure of science, and interpretations of outcomes. Nevertheless, agential realist psychology does not turn away from quantitative research but instead aspires to change former Newtonian realizations of quantitative psychology. However, first applications of agential realism into quantitative research—this paper included—can only begin initial discussions. There is still work to be done to develop a thorough understanding of the alterations of concepts, reworking of methods, and reinterpretation of findings. This work includes a further rethinking of important concepts of research that could be considered only insufficiently or not at all here. It also includes concrete tasks like revising existing methods.

### 5.1 Concrete tasks at hand

If we further elaborate on agential realist psychology, we need to aptly develop language. For European-influenced countries, Gergen and Gergen (2003) already asserted that too few good terms can describe relational thinking. This situation itself is an agential cut-enacting configuration and has its impact. Nouns imply an essence that determines why something is called what it is called. As one strategy, in English, it is sometimes possible to make a verb of a noun to indicate the enacting instead of stating a being. ‘To gender’ a person transports another meaning than ‘the gender’ of a person. Another strategy—one that Barad used frequently—is using hyphens to link words and concepts together. Like relata-within-relations emphasizes the becoming of relata through their relations, linguistic constructions like ‘feature-system’ could indicate an understanding of entanglements. Nevertheless, changing language requires agreement between more people who use a language.

Another task will be to examine previous methods. The alteration of concepts makes it necessary to examine existing quantitative methods and their suitability for agential realist conceptualizations. I suppose the knowledge of methods that can provide information about entangled configurations is growing. However, such methods are still primarily implemented to try to delete ‘unwanted influences’ instead of working with entanglements. For instance, Hanfstingl (2022) discusses the combination of ‘specification curves’ with ‘combinatorial meta-analyses’ to gain information about the effects of researchers’ decisions. Another example is the already mentioned project of Landy et al. (2020). It would be interesting to apply such methods to work *with* the entanglements as configurations that are part of the phenomenon rather than to apply such methods to be able to delete the entanglements as a disturbance from the overall picture. Furthermore, as mentioned, Pettigrew (2018) and Jost (2024) promote multilevel modeling to link different complexity levels. Agential realist psychology can learn from these methods, but it is necessary to examine them in relation to the basic assumptions of agential realism.

On the methodological side, there are already sophisticated recommendations to imply quantum probability theory (QPT) for the modeling of cognition, called quantum cognition (e.g., Pothos and Busemeyer, 2022; Busemeyer and Wang, 2015). Unlike agential realism, the quantum cognition perspective does not formulate an understanding of the ontic state of research objects or the consistency of our world but an understanding of the nature of human cognition. Quantum cognition offers a model for the working of human cognition; agential realism offers a model for the ‘worlding’ of our world. For instance, quantum cognition applies an ontic indeterminacy to decision-making processes but not to psychological research logic. Nevertheless, I am convinced that an agential realist psychology can learn enormously from handling probabilities within these approaches because the researchers install QPT calculations due to the assumed indeterminacy and not because of uncertainty about where the ‘true score’ is. So, I encourage approaching these QPT calculations of quantum cognition, not because they are a good model for human cognition but a good model for the ‘worlding’ of our world. In contrast, although item response theory (IRT) relies on probabilities, it still follows the classic understanding of the existence of a latent trait (in an ontic sense), which has to be measured in ways as sophisticated as possible (epistemologically spoken). It does not assume a core indeterminacy as part of every outcome but uses probabilities for epistemic reasons of ‘not knowing well enough’ (see differentiation in section 3).

Furthermore, developing methods to gain knowledge about the *realization potential of situated configurations* seems necessary. In mechanics, researchers might be able to repeat the same measurement process many times, but in psychology, this is far more complicated. Researchers can just let a ball hit the detection screen repeatedly to get an idea of the distribution curve of these configurations. This kind of repetition obviously will not work with persons. We might want to differentiate the realization potential of the behavior of person-system A from that of person-system B while still incorporating their overlap. Currently, a measurement is taken as an indication of the ‘true score’ with a specific uncertainty, but can that measurement be taken as an indication of the realization potential? What else can help to gain information about which realizations are less likely for person-system A than are other realizations?

In addition, there is much more to say about replication from an agential realist perspective. If we reconceptualize findings as not telling something about a ‘true score’ but about material-discursive practices, then we must continue rethinking replication. How do researchers deal with the extra variance stemming from an ontic indeterminacy until intra-actions enact agential cuts? How do researchers interpret an outcome itself as part of a realization potential when it belongs to the larger phenomenon that realization A (e.g., a group difference) sometimes happens and sometimes does not?

Hopefully, psychologists will see many more tasks at hand to elaborate further on an agential realist quantitative psychology. This paper can only start some discussions; indeed, different discussants’ backgrounds will enrich and differentiate the elaborations.

### 5.2 Further working out of conceptualizations

Other tasks are concerned with mapping out some already developed conceptualizations further. For instance, it became clear that agential realism demands the inclusion of ethical reasoning because researchers are also part of the material-discursive boundary drawing. It also became clear that for onto-epistemic reasons, we cannot distinguish between an influence from the researcher and no such influence, which deprives us of the opportunity to try to delete the former. We must learn to incorporate ethical reasoning and the researcher’s standpoints transparently and constructively. We must work out forms of assembling perspectives instead of trying to find a perspective from nowhere. Because researchers are humans, this might lead to a new psychology of science that does not see the researcher’s practices as erasable disturbances but as onto-epistemic entanglements.

Further elaboration is also required in the understanding of context as entangled relations and not as a third variable. The field of cultural psychology demonstrates how to execute this perspective not as a psychological sub-discipline but as a general perspective on phenomena (*cf.* Chakkarath and Straub, 2020). This shows some fundamental similarities to the agential realist perspective. For instance, taking embeddedness seriously means dropping essentialism concerning objects and categories. If we see the context as co-creating, we question experiments about social phenomena conducted at the computer. One task is to rework measurement designs with a fundamental inclusion of the context and the researcher’s position as entangled parts. Such acknowledgments that researchers are also embedded must move from the end of papers—where Weber et al. (2023) and Greenwald (2012) put it (see section 4.2)—to the start of research. That is, research must be built upon the premise of entanglement.

Of course, all these alterations affect the quality criteria for research. Future tasks include elaborating on quality criteria for agential realist psychology. Objectivity has already been renewed by Barad (see section 3.4). Nevertheless, researchers can use clearer instructions about communicating material-discursive practices within both psychic and psychological phenomena. Moreover, the concept of reliability has to be revised, and the concept of validity. For example, Barad does not discuss the concept of validity, hardly uses the word, and if so, then in a conventional sense of indicating something with “limited” (Barad, 2003, p. 823) or “questionable validity” (Barad, 2012, p. 12). However, validity cannot be applied to measurement in the contemporary way of quantitative psychology to describe that a test measures ‘what it aims to measure’ and that a measurement process delivers a true (as possible) representation of an entity. When measuring is instead an intra-action that can resolve the indeterminacy into a determined state, there must be a non-representationalist form of validity. This new validity has to include the idea of a ‘faithful account of a real world’ (Haraway, 1988) but does not understand measurement as the practice of relating a number to a pre-existing quantity (see discussion of Trout’s definition in section 2.2). Further discussions of an agential realist validity and reliability are needed.

This paper encourages psychologists to *reconsider what their knowledge represents*. With Barad’s agential realism, a new proposal about ‘intra-actions of natures-cultures’ emerges: “Knowledges are not innocent representations” (Barad, 1996, p. 189). “Hence, (…) what is at issue is not knowledge *of* the world from above or outside, but *knowing as part of being*” (Barad, 2007, p. 341, emphasis in the original). Knowing (Table 1) is then not to have information about the state of something. Instead, “knowing is a matter of differential responsiveness (…) to what matters” (Barad, 2007, p. 380). If I ‘know something’, I can respond differently, but not because ‘my bounded entity’ can ‘act independently’ with ‘having information.’ Rather, I can respond differently because with ‘knowing’, I am part of possible intra-actions and part of material-discursive practices. Agential realism shifts ‘knowing’ away from cognition—which is another example of why we need adapted language for these understandings. It understands practices of knowing and being as mutually implicated. To ‘know’ is kind of ‘taking part’ and also to ‘do.’ Within psychological science, we must consider how our onto-epistemical and onto-epistemological practices intra-act and co-create realizations. Many psychologists want information in the first place to make the world a better place. With agential realism, we skip the idea of ‘gaining information first’ but proceed directly to try to realize better realizations—which, as we know already, needs ethical lines to locally and temporarily define what is ‘better.’ Science (Table 1) will not detect ‘deterministic causal structures’ but will help to understand situated possibilities.

Of course, in this paper, I made agential cuts myself. Corresponding to the agential realist perspective, the aim is not to avoid those but to communicate them as well as possible. I hope this text is transparent about which line of thinking is followed at which point and where turns are taken so that colleagues can enter the reasoning and realize other or similar cuts from their perspective and entanglements. Moreover, I suppose some training is needed to consider the dimensions of agential realism. I suspect that most quantitative psychologists are trained in thinking models and language that support classic understandings. I propose we take some time to rethink and relearn, but I recommend to start now.

## Data Availability

The original contributions presented in the study are included in the article/supplementary material, further inquiries can be directed to the corresponding author.

## Footnotes

^1^I use ‘we’ in this article when discussing philosophy of science perspectives because that is the topic I offer in this paper. I use ‘researchers’ when discussing concrete consequences of such perspectives for research practices.

^2^Researchers, of course, have other responsibilities (like handling research participants well), but these are outside the scope here.

^3^This does not mean that agential realism is based on Bohr’s ‘complementarity’ (“simultaneously necessary and mutually exclusive”; *cf.* Barad, 2007, p. 415); rather, complementarity (and all its consequences) also follows from this pre-assumption of fundamental *indeterminacy* instead of a *disturbance in the measurement process*.

^4^There is also a discussion of whether there are situations where such a resolution can be undone (see Barad, 2007; Schrader, 2012), but these situations and discussions are beyond the scope of this text and could be explored elsewhere.

^5^As mentioned above, to be more precise, these authors should have used ‘psyche’ instead of ‘psychology’ because, from the quoted sentence alone, we cannot be sure what exactly is meant here, but from their text, we do know they are actually talking about the psyche.

^6^Originally named “context-embeddedness of social psychological phenomena” (Jost, 2024, p. 5).

^7^This position is posed in contrast to cross-cultural psychology, which understands context as conventionally influencing the inherent processes of the human psyche. How the cultural psychology position is applied to concrete concepts is, for instance, demonstrated by Glaveanu (2014), addressing creativity, and Salter et al. (2018), addressing racism.

^8^Significantly, Pettigrew actually means ‘psychical processes’ and not ‘psychological processes’.

^9^Similarly, Greenwald (2012) puts the insight that researchers are influenced in the final sentence instead of starting from that, though the text is titled “Scientists Are Human.” The strategy, then, is not to overthink research processes but to try even harder to overcome such influences.

^10^Other reasons for categorization include common fate or similar fit to a requirement.

^11^I suppose that this reasoning erases the distinction between ‘basic’ and ‘applied’ research, but that discussion is beyond the scope of this article and must be held elsewhere.
